# Draft genomes of three XDR *Mycobacterium tuberculosis* clinical isolates from Morocco

**DOI:** 10.1128/mra.00969-25

**Published:** 2025-12-29

**Authors:** Said Laatri, Safaa El Kassimi, El Mehdi Bentaleb, MyDriss El Messaoudi, Bouchra Belkadi, Abdelkarim Filali-Maltouf, Hassan Ait Benhassou

**Affiliations:** 1 Laboratory of Microbiology and Molecular Biology, Faculty of Sciences of Rabat, Mohammed V University 107736 , Rabat, Morocco; 2 Prevention & Therapeutics Center, MAScIR 200224, Rabat, Morocco; 3 Laboratory of Tuberculosis, Pasteur Institute of Morocco 290327 , Casablanca, Morocco; 4 MAScIR, Mohammed VI Polytechnic University (UM6P), Ben Guerir, Morocco; Queens College Department of Biology, Queens, New York, USA

**Keywords:** *Mycobacterium tuberculosis*, whole genome sequencing, extensively drug-resistance (XDR)

## Abstract

We report draft whole-genome sequences of three extensively drug-resistant *Mycobacterium tuberculosis* strains (UM29, UM64, and UM66) isolated in Morocco in 2013. These assemblies support resistance profiling, molecular epidemiology, and comparative genomics. Raw sequencing reads and genome assemblies are publicly accessible through the NCBI database under BioProject PRJNA1302428.

## ANNOUNCEMENT

Tuberculosis (TB) is a significant global health challenge, with an estimated 10.7 million cases and 1.23 million deaths reported worldwide in 2024 (1). The burden remains high in low- and middle-income countries, particularly in Africa. In Morocco, TB remains endemic, with over 25,000 new cases reported annually, and drug-resistant forms are an increasing public health concern (2). Extensively drug-resistant tuberculosis (XDR-TB), defined under the 2013 WHO criteria as resistance to isoniazid, rifampicin, any fluoroquinolone, and at least one second-line injectable agent, remains a major obstacle to global TB control (3). In Morocco, genomic data on XDR-TB remain scarce, limiting progress toward effective genomic surveillance and resistance monitoring.

To address this gap, we performed whole-genome sequencing of three retrospectively collected XDR *Mycobacterium tuberculosis* clinical isolates (UM29, UM64, and UM66) from confirmed pulmonary TB patients at the Pasteur Institute of Morocco. The XDR-TB classification of isolates was established in 2013 through routine phenotypic drug susceptibility testing using the solid-media proportion method on Löwenstein–Jensen (LJ) medium. A strain was considered resistant when ≥1% of colonies grew on drug-containing medium compared to the control (4, 5).

Sputum specimens were decontaminated using the N-acetyl-L-cysteine–sodium hydroxide method and cultured onto LJ at 37°C for up to four weeks. Colonies showing rough, buff morphology were confirmed by Ziehl–Neelsen staining, and a single, well-isolated colony was subcultured to obtain a pure strain.

Genomic DNA was extracted from a single well-isolated colony using the QIAamp DNA Mini Kit (Qiagen, Germany). Libraries were prepared using the Illumina DNA Prep (M) Tagmentation kit (Illumina, USA) and their quality evaluated on an Agilent TapeStation System (Agilent Technologies, USA). All three libraries were pooled and sequenced in one Illumina iSeq 100 run (Illumina, San Diego, CA, USA), generating 2 × 151 bp paired-end reads. In total, 3,158,814 reads were obtained for UM29, 3,348,516 for UM64, and 5,856,662 for UM66, with genome coverages of approximately 50×, 50×, and 60×, respectively. Quality control was conducted using fastp v0.23.4 (6), performing Illumina adapter sequences removal, trimming, and filtering.

Genome assemblies were generated with SPAdes v3.15.5 (7) using the—careful option, and assembly quality was evaluated using QUAST v5.2.0 (8). After filtering contigs shorter than 500 bp, the final genome assemblies had total lengths of 4,344,856 bp (UM29), 4,362,476 bp (UM64), and 4,347,274 bp (UM66), with 65.5% GC content. The assemblies consisted of 113 contigs (N50: 99 kb) for UM29, 109 contigs (N50: 97.8 kb) for UM64, and 118 contigs (N50: 97 kb) for UM66. Genome completeness ranged from 97.38% to 97.5% as estimated with CheckM v1.2.4 (9). The genome annotation was performed using the NCBI Prokaryotic Genome Annotation Pipeline (PGAP v6.10) (10), identifying 4,114, 4,130, and 4,121 coding DNA sequences in strains UM29, UM64, and UM66, respectively.

Reads were mapped to the *Mycobacterium tuberculosis H37Rv* reference genome using the MTBseq v1.1.0 (11). All isolates carried the canonical *katG* (Ser315Thr) and *rpoB* (Ser450Leu) mutations conferring resistance to isoniazid and rifampicin, respectively. Additional resistance determinants included *embB* (Met306Val) for ethambutol, *fabG1* (C-15T) for isoniazid/ethionamide, *rpsL* (Lys88Arg) for streptomycin, *rrs* (A1401G) for second-line injectables, and an ethA frameshift deletion consistent with ethionamide resistance. Fluoroquinolone resistance was explained by *gyrA* (Gly88Ala) in all isolates, with *gyrB* (Ala504Val) detected only in UM29. Default parameters were used for all software unless otherwise specified.

## Data Availability

The whole-genome assemblies generated in this study are publicly available in the NCBI database under BioProject PRJNA1302428. The corresponding GenBank accessions are GCA_052049515.1 for *Mycobacterium tuberculosis* strain UM29 (BioSample SAMN50477959), GCA_052049555.1 for strain UM64 (BioSample SAMN50478660), and GCA_052049615.1 for strain UM66 (BioSample SAMN50478818). Raw Illumina sequencing reads are available in the Sequence Read Archive under accession numbers SRR34888818 (UM29), SRR34888817 (UM64), and SRR34888816 (UM66). All datasets are publicly accessible and cross-referenced under BioProject PRJNA1302428.
